# Structural Abnormalities of the Brain Detected by 7 Tesla MRI in Patients with Usher Syndrome

**DOI:** 10.3390/jcm14186493

**Published:** 2025-09-15

**Authors:** Katarzyna Nowomiejska, Aleksandra Czarnek-Chudzik, Anna Niedziałek, Michał Toborek, Mateusz Midura, Robert Rejdak, Radosław Pietura

**Affiliations:** 1 Chair and Department of General and Pediatric Ophthalmology, Medical University of Lublin, 20-079 Lublin, Poland; aleksandra.czarnek-chudzik@umlub.edu.pl (A.C.-C.); robert.rejdak@umlub.edu.pl (R.R.); 2 Department of Radiography, Medical University of Lublin, 20-093 Lublin, Poland; anna.niedzialek@umlub.edu.pl (A.N.); michal.toborek@umlub.edu.pl (M.T.); radoslaw.pietura@umlub.edu.pl (R.P.); 3 Faculty of Electronics and Information Technology, Institute of Radioelectronics and Multimedia Technology, Warsaw University of Technology, 00-661 Warsaw, Poland; mateusz.midura@pw.edu.pl

**Keywords:** Usher syndrome, 7 Tesla MRI, visual pathway

## Abstract

**Purpose**: To analyze the structural changes in the brain related to combined hearing and vision loss in patients with Usher syndrome (USH) obtained by 7 Tesla MRI. **Methods:** Twenty patients with a diagnosis of USH and fifteen normal age- and gender-matched subjects were included in this study. USH patients underwent ophthalmological examination. All subjects underwent 7 Tesla MRI of the brain in two sequences: 3D BRAVO T1-weighted and 3D MT-weighted SILENT sequence. **Results:** Mean values of LGN volumes (right 95.65 mm^3^, left 88.61 mm^3^) are significantly (*p* < 0.001) lower in the USH group than in the control group (right 126.64 mm^3^, left 120.37 mm^3^). The average volumes of the left cuneus (4102.85 mm^3^), right parsorbitalis (2133.95 mm^3^), and right rostralanteriorcingulate cortex (ACC) (1727.60 mm^3^) in the patient group are significantly (*p* = 0.03673, 0.02434, and 0.04204, respectively) lower than in the control group (4673.73 mm^3^, 2485.13 mm^3^, and 2060.00 mm^3^, respectively). Mean lengths of the left lingual cortex (1.99 mm) and right pericalcarine cortex (1.84 mm) in the patient group are significantly (*p* = 0.02449 and 0.03153, respectively) smaller than in the control group (2.09 mm and 2.0 mm, respectively). Average lengths of the right insula (2.74 mm) in the patient group are significantly (*p* = 0.00041) greater than in the control group (2.49 mm). **Conclusions:** Parts of the brain engaged in the visual processing as LGN, pericalcarine cortex, lingual gyrus, and cuneus, are decreased, as well as those involved in hearing and language processing, such as parsorbitalis and ACC. The insula, a higher-order brain area possessing a crucial role in olfactory processing, is increased in USH patients. Our findings enhance the understanding of structural brain abnormalities related to combined hearing and vision loss and suggest complex adaptive changes that should be considered in the development of new visual rehabilitation and restitution strategies in USH patients.

## 1. Introduction

Usher syndrome (USH) is a genetically and phenotypically heterogeneous group of autosomal recessive deafness–blindness syndromes characterized by a ciliary dysfunction leading to retinal degeneration, sensorineural hearing loss, and potential vestibular dysfunction [1]. USH was first described by Albrecht von Graefe in 1858, but was later named by Charles Usher, who published a series of cases with hearing loss and retinopathy in 1914 [2]. The prevalence of USH in different populations ranges from 3.5 to 6.2 per 100,000 [3].

Both cells in the inner ear and the photoreceptor cells of the retina are known to be affected in USH and have been linked to cilia defects [4]. USH is responsible for 18% of all cases of retinitis pigmentosa (RP) in the general population, considering different ages [4] and 3–6% of childhood deafness cases, as well as approximately 50% of combined deaf–blindness cases in adults. USH is classified into three main types (Types I, II, and III), distinguished by the severity of hearing loss, the presence of balance problems, and the age of symptoms. USH types 1 and 2 are the most prevalent, accounting for 90–95% of all cases [5,6].

USH patients present progressive photoreceptor degeneration in the retina, leading to a loss of peripheral vision. Clinical symptoms include night blindness (nyctalopia) with elevated dark adaptation thresholds, abnormal electroretinogram responses, visual field narrowing, irregular retinal pigmentation including peripheral bone spicules, arterial narrowing, and optic-nerve pallor, and concomitant myopia and posterior subcapsular cataracts [7]. USH is genetically heterogeneous, with more than ten genes reported to be causative of USH [3,8,9]. The USH genes encode various proteins, including motor proteins, scaffold proteins, cell adhesion molecules, and transmembrane receptor proteins. The most common is the USH2A gene coding usherin protein, which is predominantly expressed in the retina, where it localizes to the photoreceptor cells. USH proteins have a dual function in hair cell development and mechanotransduction. There is progress toward preventing loss or restoring the function of rod photoreceptors in USH and non-syndromic inherited retinal dystrophies (IRDs). With the introduction of new molecular therapies for IRDs, there is a need for imaging modalities that can accurately detect the structural progression of the disease and for the development of reliable techniques for assessing disease progression [10].

Currently, there is no effective treatment available for USH worldwide. The hearing-loss problem can be solved by using hearing aids and cochlear implantation, but the retinal problem remains unsolved [11]. Deaf–blindness is a distinct disability that limits activities and restricts participation in society and significantly impacts an individual’s ability to communicate and navigate the world. As USH results in the loss of the two major sensory organs, the burden to patients with this disorder is tremendous. The research in USH is primarily focused on genetic causes and mechanisms [3], early diagnosis and screening, and supportive strategies; nevertheless, the data in regard to brain changes in USH patients are scarce. However, this information is required to determine endpoints in clinical trials for new treatment methods.

The use of neuroimaging allows us to visualize changes in structures in various parts of the brain, including the visual pathway, that occur under the influence of retinal disease. Tiny structures of the central nervous system, for example, LGN, previously impossible to visualize using traditional MRI, became possible to examine thanks to the use of a higher electromagnetic field and additional tools in new types of MRI. Due to high signal-to-noise ratio (SNR) values, 7 Tesla MRI allows the visualization of tiny brain structures with higher sensitivity, higher contrast, and better spatial resolution, in comparison to 3 Tesla MRI [12]. Therefore, 7 Tesla MRI has become the best tool for exploring the impact that various retinal diseases have on each element of the brain. The high spatial resolution at 7T imaging allows for accurate and possibly superior delineation of the brain structures of the central auditory and visual pathway compared to standard imaging, such as 1.5 or 3.0 Tesla MRI [8].

The aim of this study was to analyze the structural brain changes detected by 7T MRI in patients with USH.

## 2. Materials and Methods

### 2.1. Patients

USH patients were recruited at the Chair and Department of General and Pediatric Ophthalmology of the Medical University of Lublin, Poland. Approval of the Ethics Committee of the Medical University in Lublin was obtained (KE-0254/26/2020). This study was conducted in accordance with the Declaration of Helsinki. Written informed consent was obtained from all participants after a full explanation of the research procedures. Twenty patients with a clinical diagnosis of USH (mean age 32.1 years, range 17–48 years)—14 females and 6 males—have been included in this study after geno- and phenotyping (Table 1). The whole exome of each patient was targeted and sequenced. Protein-coding regions, as well as flanking intronic regions and additional disease-relevant non-coding regions, were enriched using in-solution hybridization technology and were sequenced using the Illumina NovaSeq 6000/NovaSeq X Plus system (https://emea.illumina.com/systems/sequencing-platforms/novaseq-x-plus.html, accessed on 11 September 2025).

All of the patients had hearing difficulties since childhood and were all using hearing aids. A full ophthalmological examination was performed, including visual acuity (Snellen decimal chart), optical coherence tomography (SD-OCT Topcon Maestro, Topcon Corporation, Tokyo, Japan), fundus autofluorescence, and wide-field fundus photography (Optos Daytona, Optos PLC, Dunfermline, United Kingdom). Fifteen normal subjects (mean age of 32.1 years, range of 22–44 years), including 10 females and 5 males, served as a control group.

There were no significant statistical differences between the studied group and control group (*p* = 0.8706) in regard to age and gender. Mean visual acuity in patients with USH was 0.5 (range of 0.8–0.1), whereas in the control group, it was 1.0.

### 2.2. 7 Tesla MRI Data Acquisition

For this research, MRI scans were conducted at the ECOTECH Complex in Lublin, Poland, utilizing the Discovery MR950 7 Tesla scanner (GE Healthcare, Chicago, IL, USA). The system operated with a gradient strength of 50 mT/m and a slew rate of 200 T/m/s. The dual-channel birdcage coil, driven in quadrature, was used for radio-frequency transmission, while signal reception was handled by a 32-channel array coil (Nova Head 32-channel coil, 2Tx/32Rx, Siemens, Munchen, Germany). The imaging protocol consisted of two non-contrast sequences acquired at MRI 7T: 3D BRAVO T1-weighted and a 3D MT-weighted SILENT. Detailed acquisition parameters are provided in Table 2.

### 2.3. 7 Tesla MRI Data Analysis

Quantitative analysis of brain volume and cortical thickness was carried out using FreeSurfer (version 7.4.1; Massachusetts General Hospital, Harvard Medical School; http://surfer.nmr.mgh.harvard.edu, accessed 20 June 2023), an open-source automated software suite. The “recon-all” was used for full anatomical reconstruction, which transforms high-resolution T1-weighted images into a 3D cortical model by leveraging the strong contrast between white and gray matter. This process includes standard steps such as skull stripping, intensity normalization, registration, segmentation, labeling, smoothing, and cortical parcellation. All data were processed at an isotropic resolution of 1 mm^3^. Summary statistics of segmented structures were generated using Free Surfer’s built-in tools. Volumetric analysis was based on the Desikan–Killiany atlas.

The lateral geniculate nucleus (LGN) volume was manually measured using ITK-SNAP software (version 4.0.0-rc.2), which enables the precise marking of anatomical regions of interest in three-dimensional medical imaging data. Due to the lack of access to histopathological data for the examined subjects, the true anatomical volume of the LGN could not be confirmed. Automated segmentation of the LGN was not available because of its small size, complex structure, and indistinct boundaries, which are challenging to accurately resolve in standard MR images. Evaluation of the 3D MT-weighted SILENT sequences was independently performed by three radiologists. To ensure consistency in measurements, all radiologists used identical image contrast parameters (window level: 1000; window width: 1500; minimum: 250; maximum: 1750) and made the segmentation in the same place, using the same GE Advanced Workstation and a BARCO medical-grade, calibrated monitor intended for radiological assessments. The development of the measurement protocol involved a collaborative effort among all authors, including the three radiologists, who jointly reviewed a subset of data from ten patients. This initial calibration process was essential to establish consistency and reproducibility in the manual segmentation procedure throughout the study. A sample of a typical 3D MT-weighted SILENT of the brain with left and right 3D-LGN segmentations for control and USHER syndrome-affected brain can be found at the following link: https://www.dropbox.com/scl/fi/ptaujqa4jzkvyjmwsckth/LGN-segmentation-USHER.zip?rlkey=b4zmmgokhastfhotpvm6xhxgy&st=rpt5bvul&dl=1 (assessed on 7 August 2025). 

### 2.4. Statistical Analysis

Analyses were performed in R, version 4.3.3 (29 February 2024), Angel Food Cake version 4.3.3 (R Core Team, 2024), using the dplyr and tidyr packages for data processing, ggplot2 for visualization, readxl for data import, and broom for organizing statistical model results. A significance level of 0.05 was used for statistical analyses. In this study, we present only statistically significant differences between the USH group and the control group in regard to volumetry of brain structures.

## 3. Results

**Figure 1:**
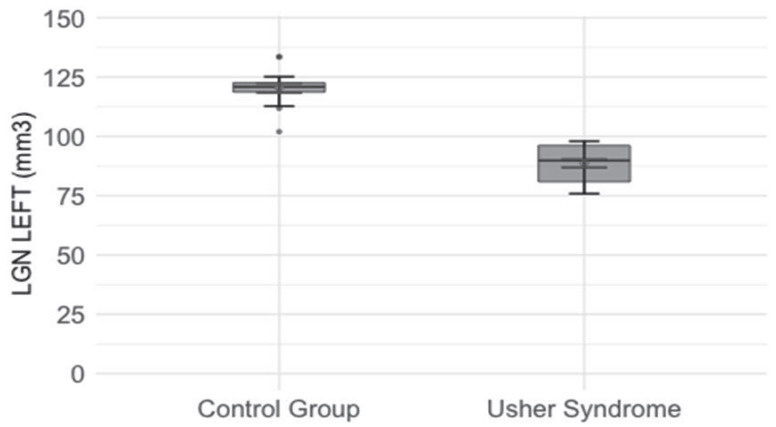
Comparison of left lateral geniculate nucleus (LGN) values between the studied and control groups. Red diamonds indicate the mean values, while blue lines represent confidence intervals. Boxplot shows the value of left LGN in the studied group and the control group. Boxes indicate the interquartile range (IQR), the black horizontal line is the median, and the black dot represents the mean. The whiskers mark the range of the normal distribution of values, and the thin horizontal dashes at their ends highlight the boundaries. Red dots indicate outliers, and blue dashes show the standard error (SE).

**Figure 2:**
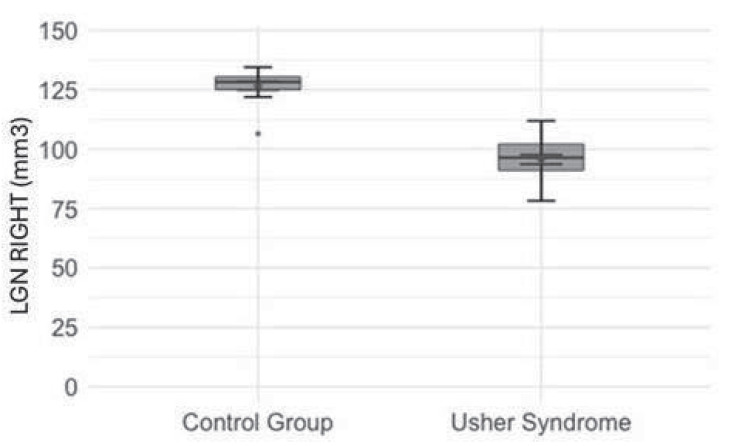
Comparison of the right lateral geniculate nucleus (LGN) values between the studied and control groups. Red diamonds indicate the mean values, while blue lines represent confidence intervals. Boxplot shows the value of the right LGN in the studied group and the control group. Boxes indicate the interquartile range (IQR), the black horizontal line is the median, and the black dot represents the mean. The whiskers mark the range of the normal distribution of values, and the thin horizontal dashes at their ends highlight the boundaries. Black dots indicate outliers, and blue dashes show the standard error (SE).

Mean left (88.61 mm^3^) and right (95.65 mm^3^) LGN volumes are significantly lower in the USH group than in the control group (left 120.37 mm^3^, right 126.64 mm^3^) (t = 11.89; *p* < 0.001 and t = 11.86; *p* < 0.001, respectively) (Figure 1 and Figure 2).

**Figure 3:**
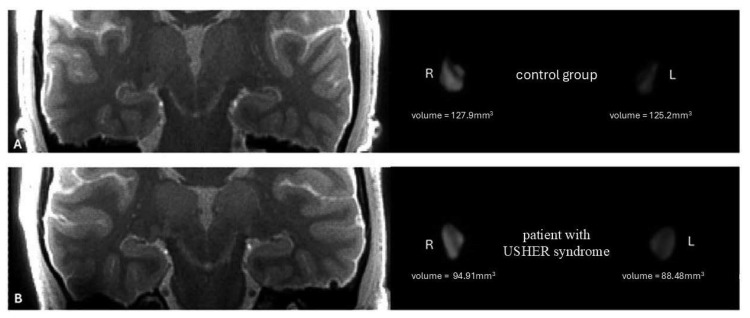
On the left—the anatomical image of the brain 3D MT-weighted SILENT with marked LGNs; on the right—3D view results of LGNs manual segmentation using ITK-SNAP software. Images show one case chosen from the control group (**A**) and patients with Usher syndrome (**B**). Images acquired at the Ecotech Complex (Lublin, Poland).

The average volumes of the left cuneus (4102.85 mm^3^), right parsorbitalis (2133.95 mm^3^), and right rostralanteriorcingulate cortex (ACC) (1727.60 mm^3^) in the patient group are significantly (*p* = 0.03673, 0.02434, and 0.04204, respectively) lower than in the control group (4673.73 mm^3^, 2485.13 mm^3^, and 2060.00 mm^3^, respectively) (representative case in Figure 3). Mean lengths of the left lingual cortex (1.99 mm) and right pericalcarine cortex (1.84 mm) in the patient group are significantly (*p* = 0.02449 and 0.03153, respectively) smaller than in the control group (2.09 mm and 2.0 mm, respectively). Average lengths (2.74 mm) of the right insula in the patient group are significantly (*p* < 0.00041) greater than in the control group (2.49 mm). Mean length of the pericalcarine cortex was significantly smaller in USH (1.84 mm) in comparison to the control group (2.0 mm). Table 3 shows all of the cortex volumes and structures with statistically significant longevity. Table 4 displays the results of test comparisons between groups, along with the *p* values.

In regard to ophthalmological examination, it was found that the LGN volume does not depend on the visual acuity and central retinal thickness obtained in OCT examination, based on Pearson’s linear and Spearman’s nonlinear correlation coefficients, and the scatter plots.

## 4. Discussion

This is the first study providing novel insights into the brain structures using ultra-high-field 7 Tesla MRI in patients with USH. The main finding of this research is that the volume of right and left LGN is significantly decreased in USH patients in comparison to controls. LGN atrophy has already been found in patients with RPGR-related RP [13], as well as in the diseases of the optic nerve as glaucoma [14,15] and Leber hereditary optic neuropathy [16]. LGN is the relay station of the visual pathway, providing the interconnection of the third (optic nerve and optic tract) and the fourth neurons (optic radiation). In a similar study of 7 Tesla MRI in 12 male patients suffering from RPGR-related RP [13], right and left LGN, as well as lingual gyrus, were significantly decreased, while the left isthmus cingulate and entorhinal cortex were significantly or almost significantly higher in the RPGR group in comparison to the control group. Moreover, the group’s thalamus-to-LGN ratio was significantly higher in the RPGR group, compared to controls. The ethorinal cortex and isthmus cingulate were more developed in RPGR-related RP patients but not in USH patients. The essential roles of the cingulate and entorhinal cortices are in consolidated memory, so the enlargement of these areas of the cortex in RP patients may be explained by the heightened need for long-lasting memory of people with decreased visual acuity. We cannot explain why these areas are not increased in USH patients, but they are not involved in vision or hearing processing.

In the current study, the pericalcarine cortex was found to be decreased in USH patients compared to normal subjects. The pericalcarine cortex centers around the calcarine sulcus and receives direct input from the thalamus, specifically the LGN. It is crucial for initial visual processing as the primary visual cortex (V1) and acts as the earliest cortical stage for visual processing in sighted individuals. Up to now, blindsight subjects showed a significant increase in pericalcarine cortical thickness [17] as a possible morphological sign of compensation underlying blindsight. However, this study examined the visual cortex of the intact hemisphere using 3 Tesla MRI of three subjects with varying degrees of cortical damage and well-documented blindsight: two with a right hemispherectomy and one with a left V1 lesion. In another study, congenitally and early blind individuals showed a thicker V1 compared to sighted controls [18]. However, in our study, patients’ visual function was quite well preserved.

In the study we conducted, the average volume of the left cuneus and the length of the left lingual gyrus in USH patients were lower than in the control group. Both the lingual gyrus and the cuneus house functional areas of V1 to V4 and play a role in basic and complex visual functions. These two gyri have been involved in both the basic and higher-order visual processing important for the orientation and direction of stimuli, color, and faces. The cuneus is a region of the brain on the medial surface of the occipital lobe, corresponding to Broadmann area 17. It plays a crucial role in the interpretation of visual signals [19]. Fibers of the superior optic radiation correspond to the inferior quadrant of the visual field; thus, lesions of the cuneus result in an inferior contralateral quadrantanopia. Adjacent to the cuneus, the lingual gyrus is involved in the processing of visual information related to memory and the recognition of objects. The tongue-like lingual gyrus is below the calcarine sulcus and represents the upper quadrant of the opposite visual field; it is considered part of cortical visual area V2. The lingual gyrus and cuneus are responsible for the initial processing of visual information received from the retina via the thalamus and receive input from the contralateral visual field [20]. Cuneus is also responsible for facilitating cross-modal, nonvisual functions, such as linguistic processing and verbal memory, after the loss of the visual senses [20].

The volume of the right parsorbitalis and right ACC in the USH group is significantly lower than in the control group. Parsorbitalis is the most rostral portion of the inferior frontal gyrus in the frontal lobe. It is responsible for the language processing network of the brain. ACC is positioned between limbic and cortical structures to integrate emotion and cognition and is thereby primed to influence amygdala-dependent learning [21]. It has already been shown in a study in rodents that the cortical circuit, from cingulate to the auditory cortex, supports auditory perceptual performance under challenging listening conditions. This pathway supports effortful listening and may be degraded by hearing loss [22]. ACC is important, as it evaluates the relevance of sensory stimuli and controls information via a descending inhibitory pathway to the thalamic reticular nucleus [23]. The thalamic reticular nucleus modulates this information flow between the thalamus and the auditory cortex by inhibiting specific thalamic neurons [24].

In our study, the volume of the insula was found to be increased in USH patients, compared to controls. The insular cortex lies deep in the lateral sulcus (Sylvian fissure), separating the frontal and parietal lobes. The insula plays a role in language and visual-vestibular integration; it also has extensive connections with neighboring brain structures, playing an important role in many critical functions of the nervous system. Increasing the volume of the insula may suggest compensatory mechanisms in response to combined hearing and vision loss. It has also already been proven in fMRI that the insula plays a crucial role in olfactory processing and activates under basic odor perception and higher-order tasks (e.g., olfactory recognition and discrimination) [25]. The pathogenesis of USH has been associated with overall ciliary dysfunction, and thus, this disease has been described as a sensory ciliopathy [1]. It is known that if olfactory receptor cells are ciliated, the hypothesis of olfactory loss in USH has emerged [26]. Moreover, the insula integrates olfactory and trigeminal information and is involved in the processing of nociceptive information [27]. Olfactory information is received by the first-order neurons in the nasal olfactory mucosa, which project to the second-order neurons of the olfactory bulb via the olfactory nerve. The olfactory bulb projects, via the lateral olfactory tract, to the piriform cortex, amygdala, and rostral entorhinal cortex. Then, these regions connect to higher-order brain areas: the orbitofrontal cortex, insula, thalamus, cingulate cortex, hypothalamus, and hippocampus [28,29]. The olfactory deficit in USH patients has been mainly evaluated by psychophysical tests and not by imaging methods. To date, 3 Tesla functional MRI has been used to assess the brains of USH patients. In the study by Ferreira and colleagues [30], the results indicated higher olfactory thresholds in USH patients, as well as decreased activity in other higher-level regions in a whole-brain approach. USH patients showed significantly increased activity in the orbitofrontal olfactory cortex when compared to the healthy controls. This region receives input from the piriform cortex (which exhibited reduced activity in USH patients). These regions were bilaterally localized and included the prefrontal cortex, insula, ventral putamen, superior frontal gyrus, and middle frontal gyrus.

In our study, we did not show the correlation between changes in MRI and ophthalmological parameters as visual acuity and central retinal thickness in OCT of USH patients. Possibly, larger studies should be performed with more parameters, such as RNFL thickness in OCT and visual field area in kinetic perimetry.

Our results support the capacity of the visual cortex to functionally and anatomically reorganize in order to support plastic changes in sensory perception. Similarly, several publications report on gray matter volume alterations due to various pathological ophthalmic conditions (e.g., age-related macular degeneration [31] and glaucoma [32]), clearly supporting the theory that non-use of cortical areas leads to remodeling and, in these cases, a reduction in neural connectivity. Schmidt and colleagues [15] described the results of 7 Tesla MRI in 20 patients with normal tension glaucoma and 16 control individuals. The LGN volume and fractional anisotropy (FA) of the optic tract and the optic radiation, and their correlation with retinal nerve fiber layer thickness, were analyzed. The conclusion was that NTG leads to significant atrophy of the LGN in comparison to controls. FA of the optic tract and the optic radiation is reduced in NTG as a sign of axonal degeneration, while RNFL thickness, but not FA, correlates with LGN volume. As LGN was decreased in USH and RPGR-related RP patients, it would also be interesting to analyze FA and RNFL in patients with USH and non-syndromic RP.

Our study has shown that parts of the brain that are part of the visual pathway, such as LGN, the pericalcarine cortex, lingual gyrus, and cuneus, are decreased, as well as those involved in hearing and language processing, such as parsorbitalis and ACC. The insula, crucial in olfactory processing, is increased in USH patients. This likely reflects degradation of some brain areas, as well as compensatory cross-modal reorganization in the absence of visual and auditory input. It is still not clear to what extent the vestibular deficits in USH patients are due to either central vestibulocerebellar or peripheral vestibular problems. Sun and colleagues [33] found that there are histological abnormalities in the vestibular hair cells of shaker-1 mice at the ultrastructural level, while the distribution of the primary vestibular afferents and the vestibular brainstem circuitries are unaffected. They concluded that the vestibular dysfunction of Usher 1B patients and shaker-1 mice is peripheral in origin.

In the future, robust methods of neuroimaging of the brain will likely play an important role in assessing the impact of eye disease on the brain and how it progresses over time. The results of the present study may provide a new tool to assess the ability of patients with retinal dystrophies to adapt to altered visual inputs. We can indicate whether the visual cortex reorganizes its function and retinotopy as a response to early retinal deficit. 7 Tesla MRI seems to be an effective tool to detect compensatory changes in the brain related to the auditory system in patients with USH. Knowledge of the impact of retinal disease on the CNS may become the basis for the construction of new possible therapeutic strategies in the future. More detailed studies are needed to correlate the results of LGN volume with elaborate retinal structure and visual function examination results. Dual sensory loss (hearing + vision) in USH likely drives distinctive remodeling across multimodal hubs in the brain. There is potential value in correlating structural 7 Tesla MRI findings with functional tests, as visual field, visual acuity, contrast sensitivity, or ERG cause reorganization and allow us to better understand brain plasticity in USH patients. Correlating structural MRI with functional/longitudinal measures turns diffuse “plasticity” into actionable biomarkers for prognosis, timing of vision restoration, and targeted rehabilitation in USH.

Structural MRI studies in IRD patients can contribute to investigating whether brain degeneration is present in patients with eye diseases. Moreover, these studies may determine the optimal timing for retinal implant insertion in the future and establish structural MRI examination as a diagnostic tool in ophthalmology. Additionally, we can expect that the adult brain has sufficient short-term plasticity to benefit from prospective therapies in IRDs.

## Figures and Tables

**Table 1:** Data of patients with Usher syndrome, including affected gene, pathogenic variant, age, gender, and visual acuity in both eyes.

Patient ID	Gene	Pathogenic Variant	Age (Years)	Gender (F-Female, M-Male)	Visual Acuity Right Eye (Snellen)	Visual Acuity Left Eye (Snellen)	Central Retinal Thickness (µm) Right Eye	Central Retinal Thickness (µm) Left Eye
1	USH2A	c.9067_9070dup; p.Leu3024Hisfs*30; deletion of exons 11–23	25	F	0.5	0.6	234	235
2	USH2A	c.2299delG; p.Glu767Serfs*21	21	F	0.8	0.8	223	221
3	USH2A	c.11864G > A; p.Trp3955*, c.2231G > A; p.Cys744Tyr	48	F	0.8	0.7	219	225
4	USH2A	c.8682-9A > G, c.3157 + 3_3157 + 5delinsTAA;	30	M	0.8	0.8	250	248
5	USH2A PDE6B	NM_206933.2c.2299del; NM_206933.2c.4714C > T	28	F	0.6	0.7	217	219
6	USH2A	c.8682-9A > G, c.3157 + 3_3157 + 5delinsTAA	31	M	0.02	0.1	203	205
7	USH2A	c.11864G > A; p.Trp3955*	22	M	0.5	0.4	210	211
8	USH2A	c.11864G > A; p.Trp3955*, c.9424G > T; p.Gly3142*	44	F	0.5	0.5	230	232
9	USH2A	c.2299delG; p.Glu767Serfs*21; c.2231G > A; p.Cys744Tyr	44	F	0.6	0.6	225	219
10	USH2A	c.638A > G; p.His213Arg VUS	28	F	0.6	0.6	220	242
11	USH2A	c.11864G > A; p.Trp3955*	44	F	0.5	0.6	230	242
12	USH2C	c.15602delT; p.Val5201Glyfs*10	22	M	0.7	0.2	217	235
13	USH2A	NM_206933.2_c.2610C > A; NM_206933.2_c.10732A > C	39	M	1.0	1.0	205	205
14	USH2A	c.8682-9A > G	36	M	1.0	0.8	262	258
15	USH2A	deletion of coding exons 21–23 c.4627 + 25435_4987 + 658de	33	F	0.7	0.8	229	232
16	USH2A	c.1663C > G, c.11864G > A c.1841-2A > G	36	F	0.4	0.5	229	230
17	USH1C	c.175_178delTTTG; p.Phe59Metfs*6	39	F	0.08	0.08	201	210
18	USH2A	NM_206933.2c.2299del: NM_206933.2c.4714C > T	28	F	0.9	0.6	225	233
19	USH2A	c.638A > G; p.His213Arg	27	F	0.9	0.3	227	246
20	USH2A	c.11105G > A; p.Trp3702*	17	F	0.8	0.8	222	218

**Table 2:** 7T MRI acquisition parameters used in imaging of the brain for this research. Abbreviations: FOV (field of view), TE (echo time), TR (repetition time), TI (inversion time), and NEX (number of excitations).

	3D BRAVO T1-W	3D MT-W SILENT
Scan duration	4 min 24 s	6 min 30 s
FOV (cm)	22 × 22	17.6 × 17.6
Slice thickness [mm]	1.0	0.8
TE [ms]	2.6	0.0
TR [ms]	6.6	257
TI [ms]	450	not applicable
Matrix size	288 × 288	224 × 2 24
NEX	1	3
Flip Angle	12	2

**Table 3:** Absolute values of different variables of the cortex in Usher syndrome (U) and control (C) groups.

Variable	Group	Mean	SD	Median	Min	Max	N
lh_lingual_mm	C	2.09	0.11	2.06	1.92	2.25	15
lh_lingual_mm	U	1.99	0.13	1.99	1.81	2.19	20
rh_pericalcarine_mm	C	2.00	0.21	2.00	1.67	2.39	15
rh_pericalcarine_mm	U	1.84	0.21	1.80	1.56	2.34	20
rh_insula_mm	C	2.49	0.17	2.44	2.22	2.80	15
rh_insula_mm	U	2.74	0.21	2.72	2.41	3.17	20
lh_cuneus_mm^3^	C	4.673.73	764.66	4.916.00	3.143.00	5.691.00	15
lh_cuneus_mm^3^	U	4.102.85	769.95	4.160.50	2.371.00	5.279.00	20
rh_parsorbitalis_mm^3^	C	2.485.13	430.30	2.517.00	1.652.00	3.297.00	15
rh_parsorbitalis_mm^3^	U	2.133.95	439.57	2.125.50	1.149.00	2.799.00	20
rh_rostralanteriorcingulate_mm^3^	C	2.060.00	474.92	1.936.00	1.341.00	2.980.00	15
rh_rostralanteriorcingulate_mm^3^	U	1.727.60	448.79	1.834.00	529.00	2.570.00	20

**Table 4:** Differences between USH and control groups using statistical tests (* means statistical significance-*p* < 0.05).

	*p* for Shapiro-Wilk’s Test		Levene’s Test		*t* Test	
Variable	Control group	Usher Syndrome	F	*p*	*t* test	*p* value
lh_lingual_mm	0.2436	0.2077	0.38	0.54241	2.36	0.02449 *
rh_pericalcarine_mm	0.9758	0.1553	0.04	0.83649	2.25	0.03153 *
rh_insula_mm	0.4957	0.8247	1.21	0.27997	−3.93	0.00041 *
lh_cuneus_mm^3^	0.5339	0.7333	0.01	0.94550	2.18	0.03673 *
rh_parsorbitalis_mm^3^	0.7313	0.6487	0.11	0.74270	2.36	0.02434 *
rh_rostralanteriorcingulate_mm^3^	0.2303	0.4874	0.04	0.83632	2.12	0.04204 *

## Data Availability

The datasets analyzed during the current study can be made available by the corresponding author upon reasonable request.
